# Long-term effects of cancer on earnings of childhood, adolescent and young adult cancer survivors – a population-based study from British Columbia, Canada

**DOI:** 10.1186/s12913-018-3617-5

**Published:** 2018-11-01

**Authors:** Paulos Teckle, Stuart Peacock, Mary L. McBride, Colene Bentley, Karen Goddard, Paul Rogers

**Affiliations:** 1 Canadian Centre for Applied Research in Cancer Control (ARCC), 675 West 10th Avenue, Vancouver, BC V5Z 1L3 Canada; 2 Cancer Control Research, British Columbia Cancer Agency, Vancouver, BC Canada; 3 School of Population and Public Health, University of British Columbia, Vancouver, BC Canada; 4 Faculty of Health Sciences, Simon Fraser University, Burnaby, BC Canada; 5 Radiation Oncology, British Columbia Cancer Agency, Vancouver, BC Canada; 6 Pediatric Oncology and Hematology, BC Children’s Hospital, Vancouver, BC Canada

**Keywords:** Childhood cancer survivors, Late effects, Long-term income, Longitudinal analysis, Health economics

## Abstract

**Background:**

The patterns and determinants of long-term income among young people surviving cancer, and differences compared to peers, have not yet been fully explored. The objectives of this paper are to describe long-term income among young survivors of cancer, the impact of socio-demographic, disease, and treatment factors on long-term income, and income relative to the general population.

**Methods:**

Retrospective cohort study with comparison group from the general population, using linked population-based registries, clinical data, and tax-records. Multivariate random effects regression models were used to determine survivor income, compare long-term income between survivors and comparators, and assess income determinants. Subjects included all residents of British Columbia (BC), Canada, diagnosed with cancer before 25 years of age and surviving 5 years or more. Comparators were selected from the BC general population matched by gender and birth year.

**Results:**

Young cancer survivors earned significantly less than the general population. In addition, survivors of central nervous system tumors have significantly lower incomes than lymphoma survivors. Survivors who received radiation therapy have significantly lower income.

Results should be interpreted with caution as the comparator group was matched by gender and date of birth.

**Conclusions:**

Depending on original diagnosis, treatment, and other characteristics, survivors face significantly lower income than peers and may require supports to gain and retain paid employment. Lower income will affect their opportunity for independent living, and will reduce productivity in the labour force.

## Background

The population of childhood, adolescent, and young adult cancer survivors has rapidly increased over the last three decades. The percentage of cancer survivors diagnosed between the ages of 0–19, who have lived for 5 years or more post-diagnosis has increased from 56% during the 1970s to 83% for cases diagnosed in the 2000s [1]. This is due to substantial progress in a multimodal of treatment regimens and improvements in supportive care [2]. However when looking at long-term outcomes of the majority of childhood cancer survivors whom are now adults even with the improved survival, they are living with late adverse treatment effects which are shown to compromise their future physical, cognitive and psychosocial health, but also their employment status [3–5].

The impact of cancer on income level and employment status has shown varying results. Previous studies have reported both negative [6–13] and neutral [14, 15] consequences of cancer on employment status. Cancer and its treatment effects have been shown to limit cancer survivors’ ability to work, compared to their labor force participation and productivity prior to diagnosis [16].

The most recent studies on labor economics emphasize the impact of employment status on health [17]. Most of the studies were conducted in the US, where health insurance is highly associated with employment status. Survivors, who are still able to work, are forced to stay active in the labor force because the implications of losing their jobs, and ultimately their health insurance plans, may be significant [18;19]. In the US, individuals who lose their employer-provided health insurance due to illness have limited alternative options to obtain insurance. In these circumstances, despite cancer-related morbidities such as physical and emotional pain, the survivor may be forced to stay employed in order to keep his or her health insurance [18, 19].

Most of the previous research on economic outcomes is focused on adult cancer survivors [16, 20], with pre-diagnosis employment. However, little is known about the employment experience and economic well-being of childhood, adolescent, and young adult cancer survivors. These individuals are at a considerably higher risk of substantial morbidity due to exposure to chronic toxicity of the treatment at a young age, prior to entering the labour force [3].

Much existing research on income and employment among cancer survivors is affected by five main methodological weaknesses. First, self-reported data are used to measure the income level of the survivors. Self-reported employment status and income level tends to be systematically mis-report, since many people are uncomfortable about disclosing their true income [21–23]. Responses may also be subject to recall bias. Second, some of the previous studies are cross-sectional in design; thus, changes in the determinants of income and employment status of cancer survivors cannot be evaluated over time [20;24]. Third, previous studies have used current or short-term household income (i.e., 6–12 months) as opposed to longitudinal income over a longer period of time [20;24]. Fourth, most studies have focused on short-term outcomes closer to the treatment phase of cancer. Finally, only a limited number of studies included a control group from the general population; as such, relative effects were not taken into account [20, 24].

The aim of this study is to examine the relationship between cancer survival and income for childhood, adolescent, and young adult cancer survivors. In this study we analyzed a unique, large, population-based set of longitudinal registries, clinical, and administrative datasets.

## Methods

### Study population

Childhood, adolescent, and young adult cancer survivors were identified from the British Columbia (BC) provincial cancer registry at the BC Cancer Agency. A survivor is defined as an individual diagnosed with cancer from the moment of dignosis for the remainder of life [25]. The sociodemographic and clinical data of the survivors were obtained from the Childhood, Adolescent, and Young Adult Cancer Survivor (CAYACS) Research Program database [26, 27]. The survivors were diagnosed between 1970 and 1999 with a first primary non-carcinoma cancer or tumor included in the International Classification of Childhood Cancer (ICCC) groups I to X for those diagnosed under 20 years [28], and the Classification for Cancers in Adolescents and Young Adults for those diagnosed aged 20–24 years [29]. Clinical data was abstracted from medical charts available from two provincial cancer treatment institutions, BC Children’s Hospital and BC Cancer Agency.

The CAYACS data were linked to the income data from Statistics Canada using personal identifiers (first name, second name, surname, gender, and date of birth). Statistics Canada stripped all identifiers and anonomyzed data were created on a secure server at Statistics Canada for analysis. Access to and analysis of the anonomyzed linked data were limited to Statistics Canada analysts. We submitted our statistical programs to Statistics Canada and directed and oversaw the analysis. Results were vetted by Statistics Canada according the Statistics Canada Privacy Act and were made available for this study.

Statistics Canada selected 10:1 control group to case cohort, matched by gender and birth date, from the BC residents in the general taxfiler file for comparison of income between cancer survivors and the general population.

## Data

### Measuring income

Income information was obtained by linking the CAYACS data to the Statistics Canada T1 Family File (T1FF). The T1FF information is based on the census family concept and incorporates household income tax and Canada Child Tax Benefit records together. T1FF also includes information from all non-taxfilers (i.e., individuals who did not file because they are unemployed or not of working age) to calculate household size and composition. The construction of the T1FF is described in Statistics Canada 2014 [30].

The main income measures were the annual individual and household income. The right-hand side control variables included sex, urban-rural status of residence and region of residence (i.e., health authority). For cancer survivors we included age at diagnosis, treatment type, and education level. As the results for individual income did not significantly differ from household income, the results for only the long-term household income are presented. We anticipate that household income is more meaningful as: (1) the cumulative long-term economic impact of cancer could only be observed with a longer observation period and (2) starting or returning to work will more likely depend on household income.

Statistics Canada contains income data items from 1982 onwards; this information is derived from annual tax records provided by the Canada Revenue Agency. To obtain a measure of income that is consistent over the years, annual income was deflated to 2010 prices using the Canadian Price Index. We generated equivalized income to adjust for household composition [31]. Equivalized income or household equivalent income was obtained by dividing the household income by a factor that is dependent on the presence of a spouse, number of dependent children, and number of other adults in the household [31–35].

### Education

The association between education and income was explored for a sub-set of our cohort of survivors (*N* = 987) for whom we have educational achievement records from the provincial Ministry of Education and Ministry of Advanced Education (Edudata). The five education levels identified are Kindergarten to Grade 12 (i.e., secondary school graduation) (K-12), developmental, non-credential, post-secondary non-academic, and post-secondary academic.

### Neighborhood measures

Neighborhood measures of urban/rural residential status and region of residence (defined according to the five regional health administrative areas or Health Authorities) were classified by geographic area using the postal code conversion file (PCCF) from Statistics Canada. Population size and socio-economic homogeneity are used by Statistics Canada to generate these geographical categories [36].

The community size information from the PCCF file defined in terms of 2006 Census population was used to assign urban-rural classifications. We grouped community size into rural Small Communities (≤10,000), Large Communities (> 10,000 – ≤ 99,999), and Metropolitan (≥100,000) [37].

## Statistical analysis

Our study used a number of econometric regression models to estimate the impact of cancer and its treatment on the income of survivors over time. Given the richness of the data with respect to the clinical characteristics, we explored the impact of cancer type, treatment type, and disease severity on the economic well-being of cancer survivors. We then investigated whether survivors experienced lower income than the general population based on demographic (i.e., sex, age, and marital status) and area-based (i.e., urban-rural classification and health authority) variables.

We explored whether an income gradient exists based on the level of education of survivors for the sub-sample of survivors we were able to link to the education database. One potential concern with our estimates for education was the direction of causality between level of education and income; it is possible that labour supply participation is affected by the level of education of the survivor as well as his or her health status. To examine this relationship, we estimated two different models, one taking education level at the time of diagnosis, which is unlikely to depend on being diagnosed with cancer, and the other model using education level at the end of the follow-up period.

We constructed four different models with the same sets of regressors. The first model (pooled linear regression (OLS)) included age, sex, age at diagnosis, treatment type, relapse, cancer site, urban-rural classification and health authority. The second model (OLS with robust standard errors) used the same regressors and calculated robust standard errors that control for heteroscedasticity.

The third and fourth models are random effects models with and without adjustment for cluster-effect. Fixed effects (FE) and random effects (RE) models are statistical analysis used for longitudinal data. The FE model assumes that time-invariant characteristics of the individuals are perfectly collinear with the person [29]. In FE model variables that do not change over time (i.e. sex, cancer site, survivor or not, level of education, urban-rural classification, and health authority) are absorbed by the intercept. The RE model assumes that the error term is not correlated with the predictors which generates an estimate for time-invariant variables. In our study we used the RE model because the key explanatory variable is constant over time (i.e., cancer survivor or not) [29].

In the model we used, a series of multivariate panel data regression models determined the relationship between survival and income. A random effects model was chosen because a fixed effects model does not produce an estimate for constant exogenous variables (i.e., sex, cancer site, survivor or not, level of education, urban-rural classification, and health authority).

The fact that we have a large individual dimension (N) relative to the time dimension (T), the random effects model was considered to be an attractive model since individual effects can be viewed as random.

We also ran the Breusch and Pagan Lagrangian multiplier (B-P/LM) test for random effects. The B-P/LM assesses whether the estimated variance of the residuals are dependent on the values of the independent variables [38]. We also calculated cluster robust standard errors which provide consistent parameter estimates in the presence of heteroscedasticity [29]. We applied the Huber/White/Sandwich VCE estimator as discussed in Arellano and Wooldridge [29, 39].

In all our models, we adjusted for age, sex, cancer type (i.e., bone, central nervous system (CNS), carcinoma, germ cell, leukemia, lymphoma, soft tissue sarcoma, and other), treatment type (i.e., chemotherapy, radiotherapy, and surgery), marital status (i.e., married, widowed, divorced, separated, and single), health authority (i.e., Vancouver Costal, Interior, Fraser, Island, and Northern), and urban-rural classification from Statistics Canada (i.e., Census Metropolitan Areas (CMAs) and larger Census Agglomerations (CAs), large communities, and rural and small towns). The analyses used Stata version 13.1 [40].

This study was funded by the Canadian Cancer Society Research Institute to utilize available data from the CAYACS program. Ethics approval for the study was obtained from the BCCA/University of British Columbia Research Ethics Board; and approvals to access, utilize and link the data were obtained from BCCA, BC Ministries of Education and Advanced Education, and Statistics Canada.

## Results

**Fig. 1:**
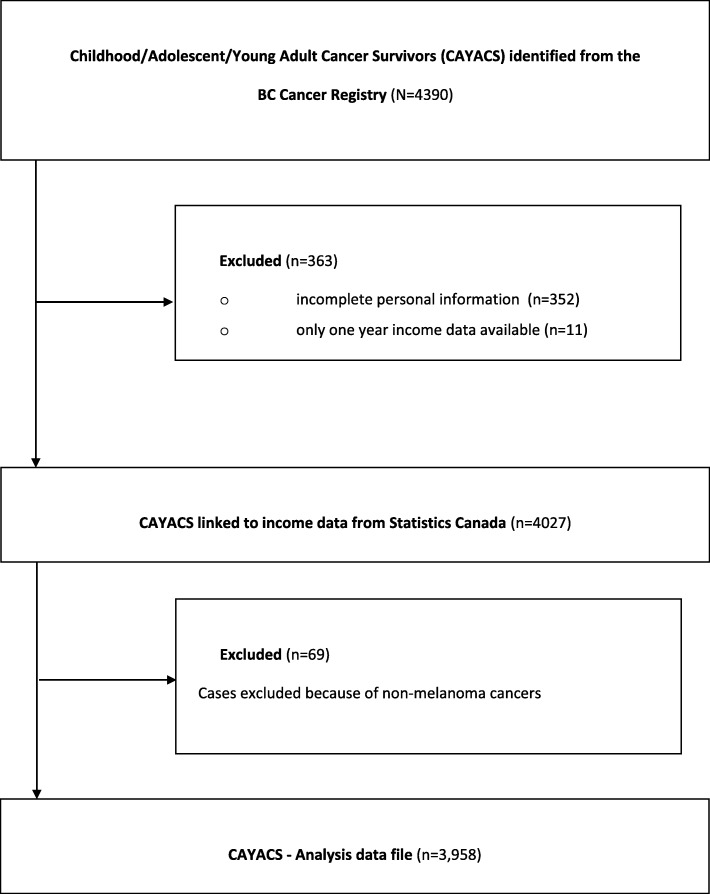
CAYACS data linked to income data from Statistics Canada

The demographic and clinical characteristics of our survivor cohort are described in Table 1. The initial cohort had 4390 cancer survivor records. Records with incomplete birth date information (*n* = 352) were discarded because they could not be linked to data in income data files, and 11 were discarded because they were linked to only one-year of tax information (Fig. 1). Of the linked cases, 2024 (51%) were males. This can be explained partly by the fact that women may be considered a loss to follow-up because they may marry into a new family name. Of the unmatched cases, 144 (41%) were males. We excluded 83 survivor records whose age was below 15 years at the time of their first tax return. Our cohort included 106,777 person-years. Table 2 compares general characteristics of the cohort and the control group. Both have similar distribution in terms of sex, being married, health authority, and rural-urban classification.

**Table 1:** Socio-demographic and clinical characteristics of survivors

	Number of Survivors (*N* = 3958)	Percent (sd)
Male	1817	49
Female	1874	51
Mean attained age in 2007	38.99	(10.00)
Mean age at Diagnosis	15.79	(7.41)
Diagnosis groups		
Bone	165	4.17
CNS	522	13.19
Carcinoma	626	15.82
Germ cell	459	11.6
Leukemia	467	11.8
Lymphoma	873	22.06
Soft tissue sarcoma	229	5.79
Other	617	15.59
Treatment type		
Chemo only	405	10.23
Radiation only	141	3.56
Surgery only	807	20.39
Other	2467	37.70
Relapsed	435	11
Education	(*N* = 987)	
K-12	76	
Developmental	2	
Non-credential Post Secondary	3	
Non-academic Post Secondary	11	
Academic Post Secondary	8	

Source: Childhood, Adolescent, and Young Adult Cancer Survivor (CAYACS) Cohort, authors’ calculations

**Table 2:** Socio-demographic characteristics of survivors and control group

	Survivors (*N* = 3958)	Control group (*N* = 20,240)
	%	%
Male	49	49
Age mean(sd)	38.9 (10.0)	39.2 (9.4)
Marital status		
Married	40	46
Widowed/separated/divorced	8	7
Single	52	46
Mean equivalized income	10.3 (0.9)	10.5 (0.9)
Urban-rural classification		
Metropolitan	39	43
Large communities	13	15
Small rural areas	48	42
Health Authority		
Vancouver coastal	21	21
Interior	17	17
Fraser	35	35
Island	17	17
Northern	10	10
Unknown	1	1

Source: CAYACS Cohort and Statistics Canada, authors’ calculations

We achieved a linkage rate of 92%. The majority of the survivors (66%) were diagnosed from age 15 to 24 years, which is 70,557 person-years. We followed survivors for 5 to 29 years with an average of 16 years. Mean age (SD) at diagnosis was 14.4 (3.7) years. Mean age (SD) at diagnosis was lower for leukemia survivors (9.8 (6.8) years) compared to all other sites (greater than 12 years). Twenty-two percent of survivors had a diagnosis of lymphoma and 20% of survivors were treated with surgery only. We generated binary categories for treatment types and 10% received only chemotherapy and 4% radiotherapy only. Most survivors attained K-12 educational level (76%). Mean log eqivalized income was lowest for CNS 10.3 (1.0) and highest for the germ cell and lymphoma 10.6 (1.0) Table 3.

**Table 3:** Mean income by cancer site

Site	Mean age at diagnosis	Mean attained age	Mean log income
Bone	15.67 (5.02)	34.97 (9.59)	10.54 (1.10)
CNS	12.80 (6.62)	33.25 (9.01)	10.38 (1.01)
Carcinoma	20.45 (3.64)	40.46 (9.87)	10.50 (1.00)
Germ cell	19.72 (5.01)	38.04 (9.40)	10.63 (1.11)
Leukaemia	9.76 (6.83)	29.90 (7.21)	10.45 (0.98)
Lymphoma	18.48 (5.09)	37.30 (9.46)	10.61 (1.00)
Soft tissue sarcoma	15.67 (7.22)	36.07 (9.93)	10.44 (1.00)
Other	16.18 (9.56)	36.79 (7.44)	10.57 (1.01)

Source: CAYACS Cohort and Statistics Canada, authors’ calculations

### Results of regression analysis

Estimates from the pooled data least squares linear regression and random effects model that includes clustered robust standard errors are presented in Tables 4 and 5.

**Table 4:** Multivariate least squares linear regression and random effects model for survivors cohort only

beta (standard error)	MODEL 1 (OLS)	MODEL 2 (RE)
Age	−0.605** (0.229)	− 0.532* (0.230)
Female	− 0.015 (0.090)	0.097 (0.096)
Female*age	−0.005* (0.002)	− 0.007** (0.003)
Age at diagnosis	0.539* (0.229)	0.468* (0.23)
RADIO ^a^	− 0.193** (0.061)	− 0.184** (0.064)
CHEMO	− 0.041 (0.039)	− 0.02 (0.043)
SURGERY	0.046 (0.03)	0.062 (0.032)
BONE ^b^	−0.084 (0.056)	−0.137* (0.067)
CNS	−0.194^§^ (0.044)	−0.176^§^ (0.045)
Carcinoma	−0.086* (0.039)	−0.086* (0.041)
Germ cell	−0.081 (0.042)	−0.091* (0.045)
Leukemia	−0.090* (0.045)	−0.104* (0.049)
Soft Tissue Sarcoma	−0.218^§^ (0.056)	−0.211^§^ (0.058)
Other	−0.044 (0.053)	−0.053 (0.054)
Relapsed	−0.068 (0.040)	−0.087* (0.040)
Small communities ^c^	−0.170^§^ (0.038)	−0.04 (0.038)
Large communities	−0.154^§^ (0.032)	−0.055 (0.033)
Interior ^d^	−0.117** (0.040)	0.024 (0.043)
Fraser	−0.018 (0.029)	0.083** (0.028)
Island	−0.119** (0.036)	−0.018 (0.044)
Northern	−0.045 (0.054)	0.077 (0.056)
Unknown	0.348* (0.137)	0.053 (0.080)
Constant	11.807^§^ (0.180)	11.516^§^ (0.190)
Sigma_u		0.706
Sigma_e		0.711
Rho		0.496
N	56,520	56,520

* *p* < 0.05, ** *p* < 0.01, ^§^ *p* < 0.001

Reference groups −^a^other treatments (included a mix of two or more therapies and no treatment): ^b^ Lymphoma; ^c^ Metropolitan; ^d^ Vancouver coastal

**Table 5:** Comparing childhood cancer survivors with control group (survivors and control cohorts)

beta (robust standard error)	Model-1 (RE)
Age	−0.033^§^ (0.001)
Female	−0.178^§^ (0.002)
Widowed^a^	−0.546^§^ (0.023)
Divorced	−0.389^§^ (0.007)
Separated	−0.512^§^ (0.007)
Single	−0.235^§^ (0.003)
Small Communities^b^	−0.234^§^ (0.004)
Large Communities	−0.118^§^ (0.004)
Interior^c^	−0.065^§^ (0.005)
Fraser	−0.028^§^ (0.003)
Island	−0.098^§^ (0.004)
Northern	0.012* (0.006)
Unknown	0.161^§^ (0.019)
Survivor	−0.089^§^ (0.011)
Constant	11.640^§^ (0.021)
sigma_u	0.214
sigma_e	0.842
Rho	0.060
N	666,480

* *p* < 0.05, *p* < 0.01, ^§^ *p* < 0.001 Reference groups −^a^ married/living common law; ^b^Metopolitan; ^c^ Vancouver coastal

We built our model starting from demographic characteristics to treatment and geographical characteristics. For the sake of brevity, we present here only the models that adjusted for socio-demographic, clinical, and geographic characteristics. In the cohort of survivors, our basic model was adjusted for sex and age at diagnosis only. Results indicated that female cancer survivors earn less than males and age was negatively associated with income (basic model not presented). When we include age and sex interaction term (to test if the association differs across age groups) and quadratic terms for age (to test a non-linear relationship), age at diagnosis is a significant (positive) predictor of income. Being a female survivor with older age had significant negative effect on income (Table 4 Model 1).

Results from the final pooled regression and random effects model that calculated robust standard errors and adjusted for clinical and neighbourhood variables showed that survivors who received radiotherapy only earned significantly less than those who received other treatments (Table 4). Survivors of CNS, carcinoma, leukaemia, and soft tissue sarcoma earned significantly less than lymphoma survivors. The diagnosis of CNS (β = − 0.176, *p* < 0.001) and soft tissue sarcoma (β = − 0.211, *p* < 0.001) were highly significantly associated with reduced income (Table 4, Model 2). After adjusting for age, marital status, and being cancer survivor, survivors living in small (β = − 0.234, *p* < 0.001) and large communities of BC (β = − 0.118, *p* < 0.001) earned significantly less than those living in large metropolitan areas (Table 5).

The Breusch-Pagan test rejected the null hypothesis of no significant differences across individuals (or variances across entities is zero) yielding a chi-square statistic of 7155(1 d.f., *p* < 0.001). The large value of the Breusch-Pagan test statistic indicates that the appropriate specification is the random effects model estimated by generalized least squares.

Results from the random effects model applied with Huber/White/Sandwich VCE estimator showed similar patterns to the pooled regression model (Table 4 Model 2). Significant negative association of radiotherapy and relapse of a disease as well as lower income by CNS and soft tissue sarcoma survivors (compared to lymphoma) were still observed in the random effects model. Germ cell survivors tend to earn significantly less than the reference group when we accounted for group variance in the random effects model (β = − 0.091, *p* < 0.05). The significant neighborhood effect observed in pooled OLS disappeared in the random effects model and we did not observe any significant difference in earnings between survivors living in large metropolitan large areas and rural areas.

Interestingly, in the random effects model, survivors who relapsed tended to earn significantly less than those who did not relapse (β = − 0.087, *p* < 0.05). Individuals from the Fraser Health Authority earned significantly more than those in Vancouver Coastal Health Authority (the reference group). Our results for the subset of survivors with qualification information indicated that survivors with a post-secondary level of education at time of dignosis had higher incomes compared to those with K-12 level of education (Table not presented). Similar association between education level at the end of follow-up of cancer survivors and income level was observed.

When comparing the survivors with our control group, females earned significantly less than males (Table 5). Individuals classified as widowed, separated, divorced or single earned significantly less than those who were married or living in a common-law relationship. Cancer survivors had a significantly lower income than the general population (*β =* − 0.089, *ρ <* 0.001). We presented our results in terms of the magnitude and direction of the association rather than exact monetary values in order to comply with Statistics Canada data disclosure rules. Both cancer survivors and controls living in small rural areas earned lower incomes compared to those in large metropolitan areas. Our results indicate that 50% of the variance is due to differences across panels, as indicated in Table 4.

## Discussion

This population-based, long follow-up study showed that childhood, adolescent, young adult cancer survivors earned significantly less than the general population of BC. Survivors of CNS tumors had a significantly lower income than lymphoma survivors. The study’s findings demonstrate that demographic and cancer characteristics significantly predict survivors’ income. As similar to previous studies utilizing self-reported income and out-of-pocket expenses [24], lower incomes were reported among cancer survivors when compared to the general population.

Our study demonstrated both sex and education levels of the cancer survivors impacted on their income. Specifically, a lower income was reported for older female cancer survivors; this finding has been reported in other studies [20, 41, 42]. This adds further support to the hypothesis that the effect of health on income is an important determinant which is more pronounced for older females. Older females remain among the most economically vulnerable groups. It is of a major concern whether older female Canadians will outlive their financial resources.

In addition, cancer survivors with higher education levels earned significantly more than those with less educational qualification. This is similar to what previous studies have found in cancer survivors [43] and in other chronic diseases like diabetes [44]. Survivors who received radiation therapy have significantly lower income compared to those received a mix of two or more treatments. Radiation therapy that directly impact nervous system may result in sensory, motor, and cognitive damage that may have adverse consequences on educational attainment and future employment. In this study a lower income was reported for CNS survivors; this is in line with results of previous studies [22, 24, 45, 46]. Neurocognitive impairment and negative emotional and psychosocial late effects, poor educational outcomes, and higher odds of unemployment after treatment for CNS tumors is common in survivors [5, 22, 24, 47–54]. The lower income observed in this study is likely to be attributed to the multiple, severe long-term health problems survivors of CNS generally experience.

The strength of our study is the long follow-up of survivors and the abstraction of treatment data from medical records. Self-reported income is used in most previous studies, which is prone to bias. This study utilized tax-reported income, which eliminates the bias of self-report.

A potential limitation associated with the use of income from Statistics Canada is the lack of information regarding job-type and hours worked; however, differences in full- vs. part-time employment would be reflected in insurance benefits that are included in annual income. Future studies should examine employment status as an outcome of interest and examine its association with income of survivors overtime.

We have matched the case and the control by sex and birth date. Urban rural classification and marital status were obtained after our data is linked to income data from Statistics Canada. We did not have prior knowledge of these variables. The distribution of cases versus control by urban rural classification is different. Survivors seem to be more likely to live in small areas and the proportion of ‘single’ in the control group is lower than the survivor group. It is important in future studies to include marital status and urban-rural classification in matching survivors to the control group.

Another limitation of the study is absence of information related to the level of education and ethnicity of subjects. We assessed the impact of education for a sub-sample of cancer survivors but not for the control group that was created from T1FF. A further potential limitation with the data from Statistics Canada, or any administrative data source, is a lack of control over data selection, quality, and collection. The prevalence of random or systematic data entry errors can be difficult to estimate; however, systematic, differential errors are unlikely, and random errors should be evenly distributed between the study groups. Additionally, subject attrition from datasets due to out-of-country migration can be problematic. However, follow-up by using the T1FF will include data from across Canada; national emigration in the relevant age groups has been low.

Finally future studies on income of cancer survivors should look at the dynamic relationship between income in adolescent versus young adult, and adult age. Dynamic panel models or trajectory analysis could provide some light into this association.

## Conclusion

Using unique linkage of the cancer cohort with the Longitudinal Administrative Data from the Statistics Canada, we estimate long-term economic impact of cancer on labor market outcomes of cancer survivors by comparing their outcomes to those of the comparison group consisting of people from the general population. Young cancer survivors earned significantly less than the general population. In addition, survivors of central nervous system tumors have significantly lower incomes than lymphoma survivors. Survivors who received radiation therapy have significantly lower income..

In light of these findings, it is vital that the Ministry of Health, cancer care institutions, labor force organizations, disability programs, and industry develop policies to address the economic status of survivors of childhood, adolescent, and young adult cancers. Particular attention should be given to those previously treated for CNS tumors and to those who received radiation therapy Economic inequality can only be associated with hardship for survivors. The development of programs that provide life-long support and rehabilitation is critical.

## Abbreviations

BC: British Columbia
BCCA: British Columbia Cancer Agency
CAYACS: Childhood, Adolescent, and Young Adult Cancer Survivor
CNS: Central nervous system
PCCF: Postal code conversion file
T1FF: T1 Family File
